# Neutrophil- and Platelet-Lymphocyte Ratio as Biomarkers of Severity in Complicated Diverticular Disease

**DOI:** 10.7759/cureus.56656

**Published:** 2024-03-21

**Authors:** Hugo Fernando Narváez González, Israel De Alba Cruz, Pabel Ruben Carbajal Cabrera, Yunuen Ailyn Morales Tercero, Luis Gerardo Luna León, Arcenio Luis Vargas Ávila

**Affiliations:** 1 Surgery, Hospital Regional "General Ignacio Zaragoza" ISSSTE, Mexico City, MEX; 2 Colorectal Surgery, Hospital Regional "Lic. Adolfo López Mateos" ISSSTE, Mexico City, MEX

**Keywords:** neutrophil-lymphocyte ratio, platelet-lymphocyte ratio, complicated diverticular disease, biomarkers, acute diverticulitis

## Abstract

Introduction: Diverticulitis is a prevalent gastrointestinal disease that may require surgical intervention. The aim of the study was to investigate the involvement of neutrophil-lymphocyte ratio (NLR) and platelet-lymphocyte ratio (PLR) as biomarkers of severity in complicated diverticular disease (CDD) in Mexican patients and their correlation with the need for surgical intervention, the length of hospital stay, and mortality.

Material and methods: An observational, longitudinal, and retrospective study performed from 2017 to 2021 was considered in patients over 18 years of age, with a diagnosis of CDD by using computed tomography and with a hemogram taken in the first 24 hours upon admission to the emergency department to describe the sensitivity, specificity, and positive and negative predictive values (PPV and NPV, respectively) of NLR and PLR in the CDD.

Results: A total of 102 Mexican patients suffering from CDD, 54% women and 46% men with a mean of 59 years, were analyzed. According to Hinchey's classification, 79 (77.5%) patients showed type I, 12 (12.8%) type II, 5 (4.9%) type III, and 6 (5.9%) type IV. The mean hospital stay was 8.8 days, with a mortality rate of 3.9%. The cut-off value was established at 5.1 for NLR according to the results of the receiver operating characteristic (ROC) curve with an area under the curve (AUC) of 0.633, a sensitivity of 90%, a specificity of 43%, PPV of 21.8%, and NPV of 96% for the prediction of CDD. A cut-off value for PLR at 72 was established according to the results of the ROC curve with an AUC of 0.482, a sensitivity of 78%, a specificity of 40%, PPV of 96%, and NPV of 9% for the prediction of CDD.

Conclusion: The NLR and PLR are easily calculable and accessible biomarkers that can be part of the decision-making for the diagnosis and treatment of CDD in Mexican people as has been observed in other populations. However, more prospective, multicenter comparative studies are needed to assess the efficacy and safety of these biomarkers in relation to those already described.

## Introduction

Acute diverticulitis (AD) is a prevalent gastrointestinal disease that may require surgical intervention by traditional laparoscopy or the novel robotic-assisted approach [1]. In the case of diverticular disease (DD) of the colon, it is a major cause of hospital admissions and a significant contributor to healthcare costs in Western and industrialized societies [2,3]. Diverticulosis affects one-third of adults older than 45 years and more than two-thirds of adults older than 85 years, of these patients, 10-25% may develop AD. Besides, AD has increased in the number of cases in young patients mainly in the Western population, and approximately 10-15% of all patients with AD present complications such as abscess, fistulae, and perforation, called complicated diverticular disease (CDD) [4-6].

To improve the diagnosis of these complications in patients with CDD, different diagnostic methods have been searched and developed. Two of them are the neutrophil-lymphocyte ratio (NLR) and the platelet-lymphocyte ratio (PLR) which are considered possible biomarkers. The rise of genomics and other advances in molecular biology such as the C-reactive protein (CRP) and calprotectin are both useful biomarkers in AD [7]. Advances in molecular biology have promoted biomarker studies as being a whole new and promising era for the timely diagnosis and treatment of many diseases. The NLR and PLR have also been considered important biomarkers for AD [8]. These ratios are obtained by dividing the absolute number of neutrophils by the absolute number of lymphocytes (NLR) and the number of platelets by the absolute number of lymphocytes (PLR). Both arise from the pathophysiology of inflammation as phase reactants, since neutrophils, lymphocytes, and platelets are blood cells that play an important role in its regulation [9]. These have been used as prognostic markers in many benign and malignant diseases such as heart disease, esophageal cancer, colorectal cancer, and hepatocellular carcinoma [8]. Despite this information, it is important to mention that in our acknowledgment, there is no previous data reported about those markers for CDD in the Mexican population.

The objective of this study was to investigate the involvement of NLR and PLR as biomarkers of severity in CDD in Mexican patients admitted at the Hospital Regional "General Ignacio Zaragoza" (HRGIZ) ISSSTE in Mexico City. A particular interest was to determine the cut-off value of the NLR and PLR for CDD in describing the sensitivity, specificity, and positive and negative predictive values (PPV and NPV, respectively) of these ratios and the correlation between NLR and PLR in CDD with the need for surgical intervention, the length of hospital stay (LHS), and mortality.

## Materials and methods

The experimental study design consisted of a retrospective cohort performed from 2017 to 2021 at the HRGIZ ISSSTE, Mexico. The inclusion criteria were patients over 18 years of age with a diagnosis of CDD by using computed tomography (CT) on admission and those patients with a hemogram taken in the first 24 hours upon admission in the emergency department. In contrast, the exclusion criteria were patients with a diagnosis of acute abdomen secondary to other causes, those without a hemogram taken in the first 24 hours of admission to the emergency department, those without clinical or radiological evidence of CDD, and those with incomplete data in the clinical record.

The study variables were age, gender, white blood cell (WBC) count, Hinchey's classification by CT report, NLR, PLR, type of treatment that was classified as conservative or intervention by surgery, LHS, and mortality. Data were collected through a retrospective review of medical records of patients admitted to the general surgery emergency department.

Statistical analysis

Categorical variables, such as gender, Hinchey's classification, treatment, and mortality, were reported as numbers and percentages. Numerical variables such as age, LHS, WBC count, NLR, and PLR were reported as mean value±standard deviation and median value in parentheses. The receiver operating characteristic (ROC) curve analysis was used to determine the NLR and PLR cut-off point for the prediction of CDD, the need for surgical intervention, LHS, and mortality. Once the cut-off point was obtained, the sensitivity, specificity, PPV, and NPV of the ratio were calculated. The analysis was performed using IBM SPSS Statistics for Windows, Version 21.0 (Released 2012; IBM Corp., Armonk, New York, United States).

## Results

​​A total of 128 patients with a diagnosis of CDD were identified, of which five were excluded for not meeting the inclusion criteria and 21 were eliminated for not having complete data in the medical record or not having clinical or radiological evidence of CDD. The demographic data are mentioned in Table 1, while the laboratory parameters are mentioned in Table 2. Only 102 patients with CDD were analyzed, ​​with a mean age of 59 years (26-90). The participants were predominantly women with 55 (54%) patients. According to Hinchey's classification, 79 (77.5%) patients were classified as I, while the remaining 23 (22.5%) were classified as II-IV. In 66 (64.7%) patients, the primary management was conservative treatment, which consisted of the administration of fluids, antibiotics, and bowel rest, while 36 (45.3%) required some type of surgical intervention, which included drainage of the abscess with either open or laparoscopic approach, intestinal resection with primary anastomosis, or resection with the creation of Hartmann's pouch and terminal colostomy. None of our patients underwent percutaneous drainage. The LHS had an average of 8.8 (3-64) days and a reported mortality of four (3.9%) individuals. The average WBC count in the 102 analyzed patients was 12.5x10^3^. The average values of NLR and PLR were 8.3 (0.5-47) and 227.72 (45.2-767.5), respectively.

**Table 1:** Demographic data for patients with CDD CCD: complicated diverticular disease, SD: standard deviation, LHS: length of hospital stay

Variables	Patients with CDD (n=102)
Mean age (±SD) (range)	59 years (26-90)
Gender (percentage)	47 female (54%)
	55 male (45%)
Hinchey's classification (percentage)	I=79 (77.5%)
	II=12 (12.8%)
	III=5 (4.9%)
	IV=6 (5.9%)
Conservative management (percentage)	66 (64.7%)
Surgical intervention management (percentage)	36 (45.3%)
Mortality (percentage)	4 (3.9%)
LHS (±SD) (range)	8.8 (3-64)

**Table 2:** Diagnostic accuracy of inflammatory markers and outcomes This table shows the results obtained from the calculation of the AUC, resulting from the correlation between the NLR, PLR, and outcomes (prediction of CDD, need for surgical intervention, and mortality). AUC: area under the curve; NLR: neutrophil-lymphocyte ratio; PLR: platelet-lymphocyte ratio; CDD: complicated diverticular disease; PPV: positive predictive value; NPV: negative predictive value

Index	Correlation between CDD and outcomes	Cut-off value	AUC	Sensibility	Specificity	PPV	NPV
NLR	Prediction of CDD	5.1	0.633	90%	43%	21.8%	96%
	Need for surgical intervention	6.4	0.790	71%	56%	57%	70%
	Mortality	7.2	0.591	80%	62%	7.8%	98.7%
PLR	Prediction of CDD	72	0.482	78%	40%	96%	9%
	Need for surgical intervention	104.1	0.557	43%	80%	89%	26%
	Mortality	120.83	0.476	3%	96%	67%	26%

The analysis of the NLR and PLR values ​​was carried out using ROC curves, to establish the determining cut-off point for predicting CDD, need for surgical treatment, and mortality, with the respective sensitivity, specificity, PPV, and NPV (Table 2). The values ​​identified as cut-off points for the NLR were 5.1, 6.4, and 7.2 for the prediction of CDD, need for surgical treatment, and mortality, respectively. The values identified as cut-off points for the PLR ​​were 72, 104.1, and 120.83 for the prediction of CDD, need for surgical treatment, and mortality, respectively. We identified that the NLR had a sensitivity of 90% and a high NPV to rule out the prediction of CDD, with a value of 96%. On the other hand, the PLR ​​had a high PPV for CDD prediction diagnosis of 96%. Conversely, the PLR ​​had a specificity of 80% and a PPV of 89% for the need for surgical treatment. In the case of mortality prediction, the PLR ​​had a high specificity of 96% to rule out CDD with a cut-off point of 120.83. The rest of the information is itemized in Table 2.

## Discussion

It is known that a diverticulum is a herniation of the mucosa and submucosa points of weakness in the colon where vessels penetrate the muscular tunic. The pathophysiology of diverticulum consists of abnormal colonic motility, which is an important predisposing factor in the development of diverticula, with segmentation contractions that separate the lumen into chambers, increasing intraluminal pressure and predisposing to mucosal and submucosal herniation [10-13].

In the last decade, there has been a reported increase in hospitalizations and healthcare costs associated with AD in the world and the economic burden of DD with more than 2.7 million annual consultations in 2010. On the other hand, according to the 2012 Nationwide Inpatient Sample Report, diverticulitis without hemorrhage was the cause of more than 216,000 admissions, with an increase of 21% when compared to the 2003 data, generating a cost of $2.2 billion US dollars. In Mexico, there are no figures in that regard [14,15]. To our knowledge, this is the first study reporting data of Mexican patients that seeks to identify both NLR and PLR as biomarkers used in the diagnosis of CDD.

Regarding the diagnosis, CT is the imaging study of choice in CDD, since it defines a therapeutic approach and/or predicts complications in patients requiring surgery by using Hinchey's classification modified by Wasvary with a diagnostic sensitivity and specificity of 94% and 99%, respectively, and a PPV of 94.3% and an NPV of 98.9% [16-19]. In our study, all patients were diagnosed with CT and reported based on Hinchey's classification.

Biomarkers have been used in clinical medicine for decades. A biomarker is an indicator of the normality of biological or pathogenic processes or pharmacological responses to a therapeutic intervention, which is objectively measured and evaluated [7]. The first biomarker was attributed to Isaakson in 1980, when he proposed using urinary nitrogen measurement as an independent marker of protein intake measurement, and it remains one of the main biomarkers used [20]. Since then, they have been used as useful markers in many types of diseases, for example, both CRP and calprotectin tests have been identified as useful biomarkers of inflammation, recurrence, and prediction of the CDD [19,21]. In a similar manner, the serum butyrylcholinesterase, another low-cost biomarker, has been used in colorectal surgery patients, with decreased levels of this biomarker in patients undergoing colorectal surgery with surgical site infection and high prognostic significance in the development of septic complications [22,23]. One of the justifications for which we carried out this study was because in our environment not all hospitals have the possibility of performing CRP in the context of DD. Therefore, the interest in the use of new non-invasive, reliable, and low-cost biomarkers for the early identification of patients with CDD is continuously required [24]. 

The NLR and the PLR are readily available, low-cost inflammatory markers [25]. Also, the benefit of the NLR has been noted in various diseases such as malignancies, coronary artery disease, acute appendicitis, acute cholecystitis, acute pancreatitis, and community-acquired infections [9,26]. The PLR has become increasingly popular as a prognostic indicator of inflammation in inflammatory and immunologically based disease, and both NLR and PLR can serve as useful biomarkers for the differential diagnosis of diverticulosis and diverticulitis and to determine the prognosis in diverticulitis patients when used in addition to other tests [26]. 

In a prospective study carried out on 101 patients of Ireland with CDD, results described an NRL above 5.34 with a sensitivity of 90.48% and a specificity of 55%. This index was attributed to a low likelihood of missing any patient who required intervention, and that study provides data comparing the accuracy of NLR to other biomarkers in the setting of AD. In addition, the authors reported an NRL with an AUC of 0.63 to predict CDD as was noticed in our study [27].

A retrospective study of 132 CDD patients carried out in Korea reported an NLR of 5.48 value for AD and 10.85 for emergent surgical intervention, with an NLR significantly higher in the emergent surgical intervention group with a statistically significant p-value (<0.0001). They also reported the delta neutrophil index (DNI), described because of the increase in the number of immature granulocytes in infection, which is better than CRP, neutrophil count, or the NLR [28]. 

In our results, a sensitivity of 90% was obtained for the prediction of CDD in patients with an NLR greater than 5.1. However, even though patients who required invasive treatment had a higher NLR, in contrast with those treated with conservative management, the sensitivity, specificity, and predictive values were inferior. In a similar manner, we did not include the DNI parameter for our analysis, but it would be a good application in the future and even a biomarker that can be compared to others such as CRP.

In a retrospective study involving 225 patients, the NLR and PLR were determined in a univariate and multivariate analysis, reporting a significant correlation between the NLR, PLR, and Hinchey's classification [29]. In the study, the NLR and PLR were reported as independent factors of severity for DD, and the average of the PLR in patients who needed surgery was 200.35 as compared to 125.79 in patients who were treated conservatively [29]. From another standpoint, we had a PLR value of 104.10 to predict the need for invasive intervention with a PPV of 89%. Hence, the value found in our Mexican population was lower compared to this study's.

Finally, in another study carried out with a total of 456 patients, higher NLR and PLR values were reported in patients with complicated disease, older age, and higher Hinchey scores, with values >5.4 and >120 for NLR and PLR, respectively [25]. However, in contrast to our findings, they had lower investment rates (7.5%) compared to ours (45.3%). 

Limitations in the study

One of the disadvantages of this study was its retrospective nature, as there could be missing or incomplete data. Second, unfortunately, our institution lacks CRP for emergency use, which does not allow us to make a comparison between the effectiveness of NLR and PLR. Third, despite having taken the laboratories and the CT in the first 24 hours of admission as reported in this study, the onset of symptoms and the arrival to the emergency were not considered as well as the comorbidities, which is why some chronic diseases can modify inflammatory parameters. Preferably, a prospective and multicentric study should be carried out for better evaluation of these variables in order to increase the sample of the evaluated patients.

Ultimately, taking these indices into account may allow more precise clinical decisions to be made in other institutions in Mexico, where CT or other biomarkers already described in the literature with diagnostic validity may not be widely available, and consider these indices as another tool for the diagnosis of patients with CDD.

## Conclusions

Current data have mentioned an increase in the number of cases of DD in the world population. Due to this, there has been an increase in interest in the use of biomarkers to improve diagnostic accuracy. Our result in Mexican patients supports that NLR and PLR are easily calculable and accessible biomarkers that can be useful as a complement in decision-making for the timely diagnosis and treatment of patients with CDD. However, more prospective and multicenter comparative studies are needed to reinforce the efficacy and safety of these biomarkers.
